# Comprehensive assessment of alternative splicing analysis methods for single-cell RNA-seq

**DOI:** 10.1016/j.isci.2026.116090

**Published:** 2026-05-22

**Authors:** Quanlong Jiang, Guicai Li, Lingyan Xing, Dongmei Zhang, Junjie Sun

**Affiliations:** 1 Medical Research Center, Nantong First People's Hospital, Southeast University, Nantong, Jiangsu, China; 2 Key Laboratory of Neuroregeneration of Jiangsu and Ministry of Education, Co-Innovation Center of Neuroregeneration, NMPA Key Laboratory for Research and Evaluation of Tissue Engineering Technology Products, Nantong University, Nantong, Jiangsu Province 226001, P.R. China; 3 Medical Research Center, Affiliated Hospital 2 of Nantong University, Nantong 226001, People's Republic of China; 4 Jiangsu Provincial Medical Key Discipline (Laboratory) Cultivation Unit of Immunology, Nantong First People's Hospital, Nantong 226001, People's Republic of China

**Keywords:** Biological sciences

## Abstract

Alternative splicing expands transcriptomic and proteomic diversity and contributes to cell identity and function. Advances in single-cell RNA sequencing enable transcriptome profiling at cellular resolution, yet their performance across biologically relevant tasks has not been systematically evaluated. Here, we benchmark six representative alternative splicing analysis methods across multiple full-length, short-read scRNA-seq datasets, focusing on biologically relevant tasks including cell clustering and differential splicing detection. Among these methods, scQuint achieves robust performance across datasets and tasks. Application of scQuint to stem cell differentiation and tumor-associated immune cell datasets identifies cell type-specific splicing programs with biological significance. These results provide guidance for method selection and highlight the value of single-cell splicing analysis for uncovering regulatory mechanisms beyond gene expression.

## Highlights

• Systematic benchmark of six scRNA-seq splicing analysis methods
• ScQuint achieves top performance in clustering and differential splicing detection
• ScRNA-seq splicing analysis detects biologically relevant splicing events

## Graphical abstract

**Figure:**
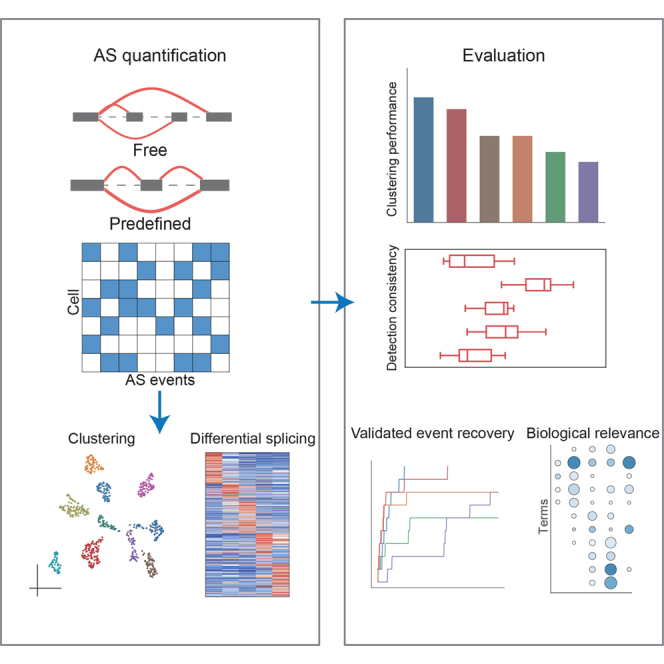

## Introduction

Alternative splicing (AS) is a post-transcriptional mechanism by which different splice sites are selected during precursor mRNA processing, resulting in the inclusion or exclusion of specific exons and producing multiple splice isoforms from a single gene. This process greatly enhances proteomic diversity and functional complexity in eukaryotic cells. AS is highly prevalent, with approximately 95% of human multi-exon genes undergoing AS.^1^ It plays essential roles in maintaining cellular homeostasis and is involved in nearly all major biological processes, including growth, development, and aging.^2^^,^^3^ Aberrant splicing contributes to various diseases, such as neurodegenerative disorders, autoimmune conditions, and cancers.^4^^,^^5^^,^^6^ Targeting mis-splicing has emerged as a promising therapeutic strategy, with several AS-modulating drugs already approved for clinical use.^7^^,^^8^ Accurate identification of AS events is thus critical for understanding biological regulation and disease mechanisms.

Currently, AS is primarily studied using bulk RNA sequencing (bulk RNA-seq), which has revealed widespread species- and tissue-specific splicing patterns and identified numerous functionally relevant AS events.^3^^,^^9^^,^^10^ For instance, our previous study demonstrated increased AS activity during skeletal muscle atrophy, where enhanced inclusion of conserved exons in the titin-interacting RhoGEF *Obscn* may contribute to muscle wasting.^11^ Integrative analyses of disease-associated bulk RNA-seq datasets have also uncovered extensive AS dysregulation. In hepatocellular carcinoma, splicing alterations in hepatocyte-specific kinases were significantly associated with patient survival and recurrence.^12^ Despite these advances, bulk RNA-seq cannot resolve cell-type-specific splicing patterns or disentangle the contribution of individual cell populations to observed transcriptomic changes.

In contrast, single-cell RNA sequencing (scRNA-seq) overcomes the averaging limitations of bulk RNA-seq by enabling transcriptomic profiling at single-cell resolution. Contemporary scRNA-seq technologies can be broadly classified into two categories: plate-based and droplet-based methods. Plate-based approaches, such as Smart-seq, Smart-seq2, and Smart-seq3,^13^^,^^14^^,^^15^ provide full-length transcript coverage, making them particularly suitable for direct analysis of AS events. In contrast, droplet-based platforms, including 10× Genomics, offer substantially higher throughput through cell barcoding but sacrifice read depth and coverage. These methods typically capture exon–intron junctions near the 3′ or 5′ ends of transcripts, depending on the library preparation protocol.^16^ Although third-generation long-read sequencing technologies enable direct observation of full-length isoforms, current single-cell long-read datasets differ substantially from short-read scRNA-seq data in data characteristics and analytical focus.^17^ Several studies have demonstrated the feasibility and value of analyzing AS at single-cell resolution. For example, splicing patterns have been profiled in neurons, pancreatic islet cells, and peripheral blood mononuclear cells (PBMCs) using Smart-seq-based data, revealing isoform-level differences between cell types and shedding light on functional specialization.^18^^,^^19^^,^^20^^,^^21^ These studies have shown that AS contributes meaningfully to cell identity and can complement gene expression in characterizing cell states. Despite these advances, single-cell AS remains an underexplored area compared to gene expression. Most current studies are limited in scale or focused on specific systems, and there is a lack of systematic analysis across diverse cell types and biological contexts. This highlights both the importance and the urgency of developing robust tools and frameworks to better understand splicing regulation at single-cell resolution, despite the unique challenges posed by single-cell transcriptomic data.

A major limitation in performing AS analysis at the single-cell level lies in the inherent challenges of scRNA-seq data, particularly its sparsity and high dropout rates.^22^^,^^23^ When read depth is low, percent spliced in (PSI) estimates become highly variable due to limited read support at splice junctions. Since PSI is computed as the ratio of inclusion-supporting reads to all reads spanning the event, it is particularly sensitive to dropout and noise.^24^ Unlike gene expression, where missing values can often be interpreted as zero, splicing ratios reflect relative isoform usage and cannot be imputed in the same manner. These limitations make accurate PSI estimation in single cells particularly difficult. To address these challenges, a number of computational methods have been developed for quantifying AS in scRNA-seq data, each adopting distinct strategies to define and measure splicing events. BRIE2 employs a Bayesian framework to estimate PSI values under low read coverage.^25^ scQuint aggregates splicing information across all cells to compute a global PSI.^26^ SCASL applies a weighted k-nearest neighbor approach for PSI imputation,^27^ while MARVEL approximates PSI distributions using a random sampling strategy.^28^

Although multiple methods have been proposed for single-cell splicing analysis, a systematic comparison of their performance has been lacking. To address this, we performed a comprehensive benchmarking of six current state-of-the-art methods: BRIE2, scQuint, Psix,^29^ SpliZ,^30^ MARVEL, and SCASL. Their performance was evaluated across multiple datasets and biological tasks, focusing on two central goals in splicing analysis: clustering cells based on splicing profiles and identifying differential splicing between conditions. Beyond benchmarking, we also explored the biological relevance of the detected events and identified many cell type-specific splicing patterns, providing insights into cell-specific regulation and potential functional roles of AS. In this study, we focus on full-length, plate-based scRNA-seq datasets generated using Smart-seq-based protocols, which are among the most widely used platforms for single-cell AS analysis.

In conclusion, we found that scQuint consistently achieved the best performance in both cell clustering based on splicing profiles and the detection of differential AS events. Using scQuint, we further identified numerous biologically relevant splicing events during stem cell differentiation and within tumor-infiltrating immune cells. These results underscore the power of single-cell splicing analysis in uncovering regulatory programs that are not apparent from gene expression data alone.

## Results

### Benchmarking alternative splicing analysis methods in scRNA-seq data

**Figure 1:**
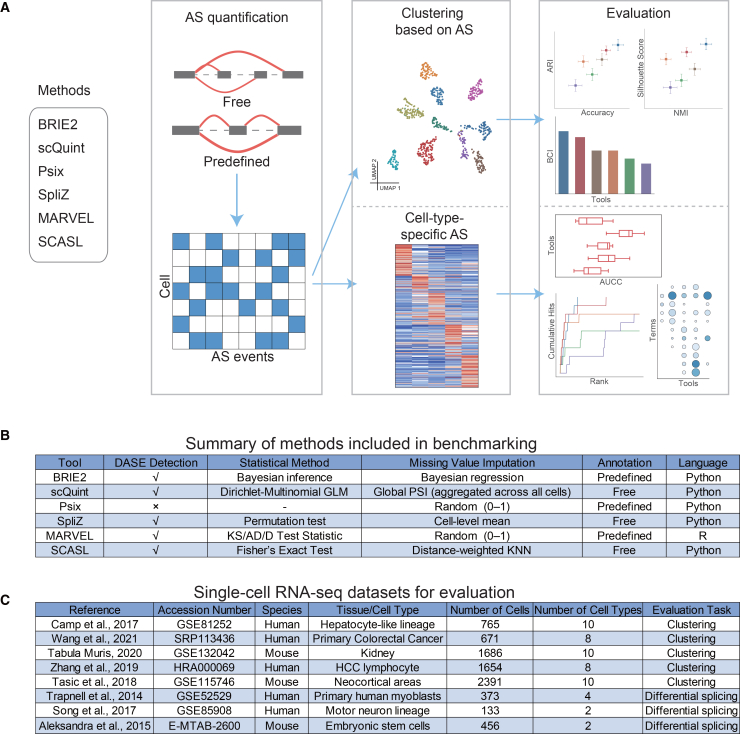
Overview of the benchmarking framework and datasets (A) Schematic of the benchmarking workflow for six computational methods designed to analyze alternative splicing in single-cell transcriptomes. Methods were evaluated based on their performance in cell clustering and in detecting differentially regulated alternative splicing events. (B) A brief overview of the methods included for benchmarking. (C) Overview of the eight single-cell RNA-seq datasets used for evaluation.

We surveyed the literature for methods developed for AS analysis at the single-cell level. We excluded those that do not provide cell-level splicing estimates, detect only junction or transcript abundances without estimating splicing ratios, or require additional spike-in controls (Methods). In this benchmarking study, we evaluated six computational methods specifically designed for the analysis of AS in scRNA-seq data, including BRIE2, scQuint, Psix, SpliZ, MARVEL, and SCASL (Figure 1B). Among these, five support differential alternative splicing (DAS) analysis, while Psix does not. The methods can be broadly grouped into predefined-annotation and annotation-free approaches. Predefined-annotation methods, including BRIE2, Psix, and MARVEL, rely on reference transcript annotations to detect and quantify splicing events. BRIE2 uses a Bayesian framework to estimate PSI values under low coverage, focusing specifically on skipped-exon (SE) events. Psix also targets SE events but calculates raw PSI values without correcting for dropout. MARVEL supports a broader range of splicing types, such as SE, intron retention, and alternative splice site usage, providing more comprehensive splicing profiles. Annotation-free methods, including scQuint, SpliZ, and SCASL, identify both known and novel splicing events independently of existing transcript annotations. scQuint detects alternative introns that share a common 3′ splice site and imputes missing PSI values using a global average across all cells. SCASL detects splicing at both the 5′ and 3′ splice sites and uses a weighted k-nearest neighbor approach for PSI imputation. SpliZ summarizes intron usage variability at the gene level to represent splicing dynamics.

We collected single-cell Smart-seq2 data from eight studies (Figure 1C), of which five were used for clustering analysis to evaluate whether AS-derived features could accurately resolve cell identity. We selected data from a single donor in Zhang et al., 2019,^31^ kidney tissue in the Tabula Muris, 2020 dataset,^32^ and randomly selected 10 distinct cell types from the Tasic et al., 2018 dataset.^33^ Five datasets from three studies were used for DAS analysis, all of which had matched bulk RNA-seq data. Detailed information on these subsets can be found in Table S1. The numbers of mapped reads and junction reads per cell for all datasets are shown in Figures S1A–S1C.

To assess the performance of these methods for single-cell RNA splicing analysis, we focused on two core tasks: (1) clustering cells based on splicing-derived features, to evaluate the ability of splicing features to delineate biologically meaningful cell types, and (2) detecting DAS events (DASE) between biological conditions. For clustering evaluation, we employed four complementary metrics: accuracy, adjusted rand index (ARI), normalized mutual information (NMI), and silhouette score. For all four metrics, higher values indicate better alignment with the ground-truth labels and more accurate clustering results. Overall performance was summarized in a biologically meaningful way by introducing the biological clustering integrity (BCI) score, defined as the unweighted average of accuracy, ARI, NMI, and silhouette score. The BCI score reflects both the agreement with known cell types and the structural quality of clusters. For the performance evaluation of DASE detection, we used three complementary criteria. First, we quantified the concordance between DASEs identified from matched bulk and scRNA-seq datasets using the area under the concordance curve (AUCC).^34^^,^^35^ Second, we evaluated the ability of each method to recover experimentally validated splicing events. Third, we assessed the functional relevance of detected events by analyzing the enrichment of gene ontology (GO) terms associated with the biological conditions.

### Performance evaluation of splicing-based clustering

We first evaluated clustering performance based on splicing-derived features across six AS analysis methods for scRNA-seq data: BRIE2, scQuint, Psix, SpliZ, MARVEL, and SCASL. To this end, we curated five benchmark single-cell datasets comprising a total of 7,167 cells spanning 46 distinct cell types (Figures 1C and S2A), which served as ground truth. Clustering performance was assessed using four metrics, accuracy, ARI, NMI, and silhouette score, as well as the overall performance metric, the BCI score. To mitigate the influence of hyperparameter settings on clustering outcomes, we conducted a parameter sweep over the number of variable events, the number of PCA components, and the clustering resolution. The optimal combination was selected based on the BCI score. (Table S2).

**Figure 2:**
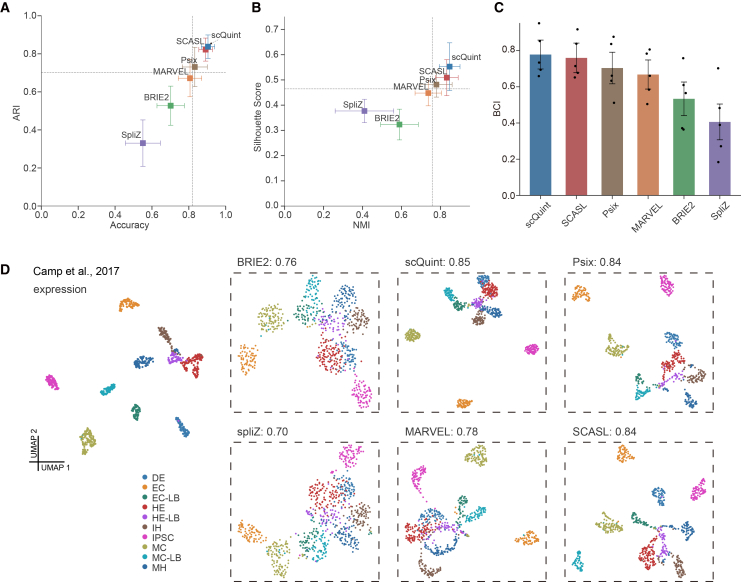
Performance of splicing-based clustering across AS methods (A) Mean accuracy and ARI scores for six methods on five scRNA-seq datasets. Dashed lines mark the median performance across methods. Values are mean ±0.5 × SD; error bars show SD across datasets. (B) Same as A, but evaluated using average NMI and silhouette scores. (C) Aggregate performance quantified by BCI scores (mean ±95% CI, *N* = 5). Each dot represents one dataset. (D) UMAP embeddings generated from AS profiles of the Camp et al., 2017 dataset illustrate the discriminative ability of each method. Clustering based on gene expression is shown as a reference. The score displayed next to each method indicates its corresponding BCI score. Cell clusters were labeled and colored by cell type annotations: iPSCs (induced pluripotent stem cells), DE (definitive endoderm), HE (hepatic endoderm), IH (immature hepatoblast-like cells), MH (mature hepatocyte-like cells), EC (endothelial cells), MC (mesenchymal cells), LD (3D liver bud).

scQuint and SCASL exhibited outstanding results, with the highest average accuracy (0.89/0.88), ARI (0.82/0.81), NMI (0.85/0.83), and silhouette score (0.55/0.51). Their average BCI score was 0.78 and 0.76, respectively, better than MARVEL (0.67), BRIE2 (0.53), Psix (0.70), and SpliZ (0.41). We used the Camp et al., 2017 dataset to visualize clustering outputs, providing an intuitive comparison of how well each method separates cell types. UMAP embeddings (Figure 2D) show that scQuint, SCASL effectively capture underlying cell-type distinctions, producing clustering patterns comparable to those based on gene expression profiles. Psix also performed well on this dataset, achieving a BCI score of 0.84. scQuint and SCASL show the highest BCI scores across all benchmark datasets (Figure S2B) and maintain strong performance even when cell types are not easily separated based on gene expression, as demonstrated in the Wang et al., 2021 and Zhang et al., 2019 datasets (Figures S3A and S3B).

Both scQuint and SCASL are annotation-free splicing methods that identify splicing events based on shared splice sites. Specifically, scQuint measures the usage of each junction by comparing it to other junctions that share the same 3′ splice site, while SCASL considers junctions that share either the same 3′ or 5′ splice site. Notably, SCASL detects nearly twice as many events as scQuint, likely due to its use of both splice site ends (Figure S2C). Among all evaluated tools, these two identify a greater number of splicing events while maintaining lower proportions of missing values (Figures S2C–S2E). Given the large variation in the number of detected splicing events across different methods, we retained the top 5,000 events with the lowest missing value ratios to mitigate the impact of varying event counts across methods. scQuint and SCASL exhibited substantially fewer missing values compared to other methods (Figure S2E), indicating their ability to produce dense, high-coverage splicing matrices with consistent event detection across a wide range of cells. In comparison, BRIE2 detected the fewest events (Figure S2C), and SpliZ had the highest missing value ratio (Figure S2E). Overall, scQuint outperformed SCASL and the other methods in distinguishing cell types and achieving accurate cell classification, performing best in our benchmarking analysis. Additionally, the splicing-based features from these methods reliably capture biological heterogeneity at the cell-type level.

### Performance evaluation of DASE detection

To systematically evaluate the performance of methods for detecting DASEs in scRNA-seq data, we used real scRNA-seq datasets from experimental studies, enabling performance assessment under realistic biological and technical conditions. Matched bulk and scRNA-seq datasets from the same purified cell populations were used to provide a reliable ground truth for benchmarking (Figure 2C). These datasets, generated in the same laboratory using consistent protocols, were profiled in parallel on biologically matched samples. Through an extensive literature search, we identified five “gold standard” datasets that met these criteria and were selected for benchmarking.

**Figure 3:**
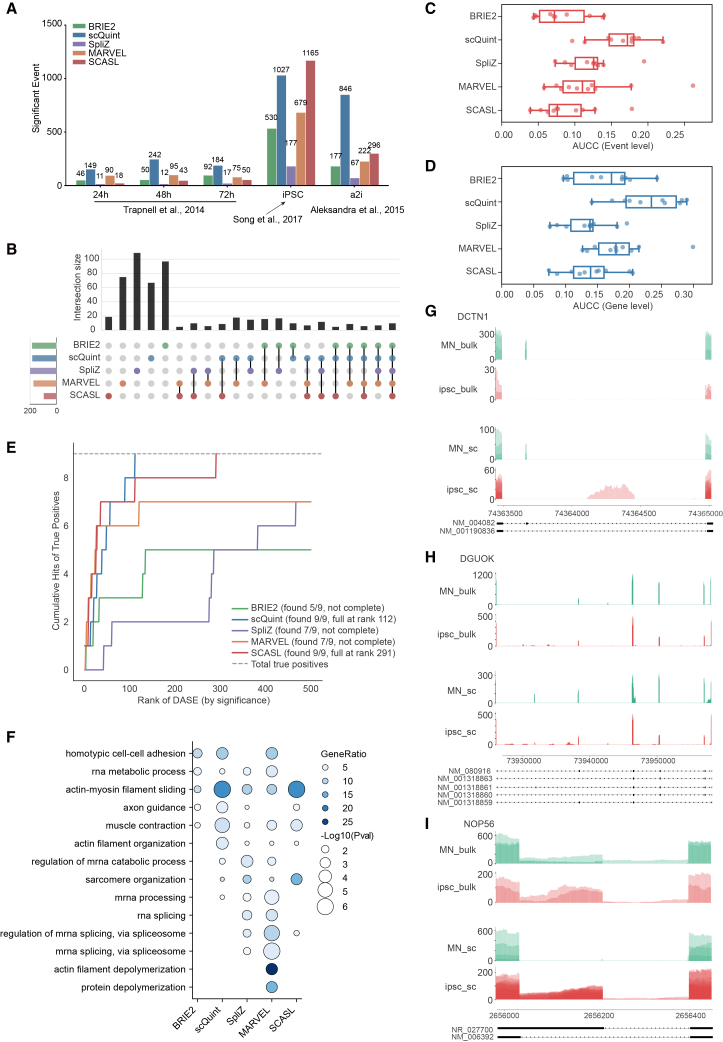
Performance of DASE detection (A) Bar plots showing the number of significant DASEs identified by each method, with adjusted *p* value <0.05. (B) Upset plot showing overlaps among the top 200 ranked splicing events for each method (Song et al., 2017 dataset). Ranking was based on statistical significance without applying adjusted *p* value thresholds. (C and D) AUCC for the top 200 ranked splicing events per method, evaluated at the event level (C) and gene level (D) across matched bulk and single-cell datasets. Boxplots show median (center line), interquartile range (box), and 1.5× IQR (whiskers). Points represent individual datasets. (E) Cumulative recall curves showing the recovery of nine experimentally validated differential splicing events by each method. The top 500 differential splicing events per method were ranked by statistical significance (*x* axis: rank 1–500; *y* axis: cumulative true positives). Methods that recover all validated events with fewer top-ranked predictions demonstrate stronger early recall. Validated events were sourced from Song et al., 2017. (F) GO enrichment analysis based on the top 200 ranked splicing events per method. Each bubble represents a GO term enrichment result, with the size indicating enrichment significance (−log_10_*p* value) and color intensity representing the gene ratio. Analysis was based on the 72-h serum-induced myoblast differentiation from the Trapnell et al., 2014 dataset. (G–I) Sashimi plots showing example alternative splicing events for *DCTN1* (G), *DGUOK* (H), and *NOP56* (I) from Song et al. 2017. For each gene, splicing patterns are visualized in MNs and iPSCs conditions. Bulk RNA-seq data (top) and single-cell RNA-seq data (bottom) are shown for comparison. Sashimi plots were generated directly from BAM files to visualize splicing patterns, and the displayed events were detected by both rMATS and LeafCutter.

scQuint identified the largest number of DASEs in both the Trapnell et al., 2014 and Aleksandra et al., 2015 datasets, and ranked second in the Song et al., 2017 dataset, narrowly behind SCASL (Figure 3A). To compare the results across methods, we examined the overlap of DASEs at the gene level (Figure 3B). scQuint and SCASL identified the fewest number of unique genes (67 and 19, respectively), while SpliZ detected the most (109). The total number of splicing events detected by single-cell and bulk RNA-seq methods is shown in Figures S4A and S4D. We next evaluated each method’s performance of DASE detection by measuring the concordance between scRNA-seq and matched bulk RNA-seq results, quantified by the AUCC. Notably, scQuint achieved significantly higher AUCCs at both the event and gene levels compared to other methods (Figures 3C and 3D), indicating higher accuracy in detecting DASEs from single-cell data. Similar trends were observed when considering the top 100 events or genes (Figures S4E and S4F).

Beyond the concordance-based evaluation, we assessed each method’s sensitivity by testing its ability to recover known splicing events validated in Song et al., 2017, which served as an experimentally grounded benchmark. We evaluated whether these validated events were ranked among the top 500 DASEs identified by each method (Figure 3E). scQuint successfully recovered all nine validated events within its top 112 ranked DASEs, while SCASL did so within its top 291. In contrast, BRIE2, SpliZ, and MARVEL recovered only 5, 7, and 7 of the validated events, respectively, within their top 500 predictions.

To evaluate the functional relevance of identified DASEs, we performed GO enrichment analysis using two representative datasets. In the Trapnell et al., 2014 dataset (72-h low-serum-induced myoblast differentiation), DASEs identified by each method were enriched in 14 pathways related to mature myocyte function, including muscle contraction, actin filament organization or depolymerization, and sarcomere organization (Figure 3F). In the Song et al., 2017 dataset, which compares motor neurons (MNs) and induced pluripotent stem cells (iPSCs), enriched pathways included cell cycle regulation, chromatin remodeling, microtubule dynamics, DNA replication, and RNA processing (Figure S4G). These results suggest that DAS is tightly linked to the core biological functions of distinct cell types. Regarding the number of functionally enriched pathways, MARVEL showed the broadest coverage, followed by scQuint and SpliZ, while BRIE2 and SCASL identified relatively fewer. With respect to concordance with known cell-type-specific functions, scQuint showed the highest functional relevance, followed by MARVEL, while SpliZ, BRIE2, and SCASL were less functionally aligned (Figures 3F and S4G).

Sashimi plots were used to visualize the dynamics of AS during the differentiation of iPSCs into MNs. The results demonstrate that the trends in AS events identified through both scRNA-seq and bulk RNA-seq were largely consistent (Figures 3G–3I). Exon skipping and inclusion were the most common forms of AS observed. Specifically, during iPSC differentiation, we noted a significant increase in the inclusion of exon 27 of the *DCTN1* gene (Figure 3G). *DCTN1* encodes p150 Glued, the largest subunit of the dynactin complex, which is involved in intracellular cargo transport and neurite outgrowth by directly binding to microtubules and cytoplasmic dynein. Mutations in *DCTN1* are associated with MN diseases, such as amyotrophic lateral sclerosis (ALS) and spinal and bulbar muscular atrophy (SBMA).^36^ Studies have shown that AS enables *DCTN1* to encode over 10 protein isoforms with varying microtubule-binding capabilities.^37^ Notably, full-length isoforms of DCTN1 are specific to mature neurons, where they contribute to microtubule stability during the formation of neurite protrusions,^38^ which aligns with our findings.

Similarly, *DGUOK*, which encodes deoxyguanosine kinase responsible for purine deoxyribonucleoside phosphorylation in the mitochondrial matrix, underwent significant splicing changes during iPSC differentiation into MNs. *DGUOK* mutations have been shown to impair mitochondrial DNA synthesis in neurons, disrupting mitochondrial homeostasis.^39^ In our analysis, we observed that *DGUOK* shifted from multi-exon-skipping transcripts to full-length transcripts during differentiation, regardless of whether scRNA-seq or bulk RNA-seq data were used (Figure 3H). Although the precise functional differences between these isoforms remain unclear, this transition could be related to mitochondrial morphological remodeling and changes in energy metabolism during differentiation.^40^ Moreover, when analyzing alternative 3′ splice site (A3SS) events, scRNA-seq analysis demonstrated the ability to match the results of bulk RNA-seq, further supporting the utility of scRNA-seq in capturing AS events at single-cell resolution (Figure 3I).

### Exploring biological insights through single-cell AS analysis

Next, we investigated whether single-cell AS analysis using scQuint could provide biological insights. We selected two datasets for this purpose: the Camp et al. 2017 dataset, which captures the hepatic differentiation lineage under 2D culture conditions, including iPSCs, definitive endoderm (DE), hepatic endoderm (HE), immature hepatoblast-like cells (IH), and mature hepatocyte-like cells (MH). The Zhang et al. 2019 dataset includes five common leukocyte types: CD4^+^ T cells, CD8^+^ T cells, natural killer cells, mo novelcytes, and macrophages. The scQuint BCI scores for these datasets were 0.85 and 0.63, respectively (Figures 2D and S3B).

**Figure 4:**
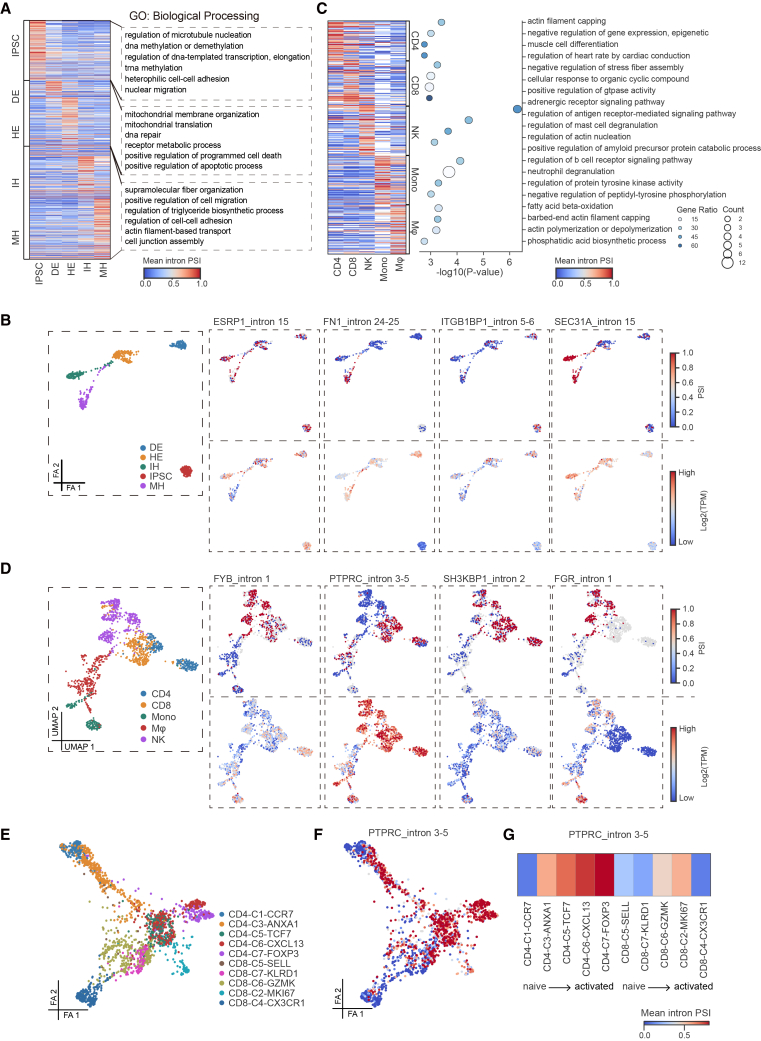
Single-cell resolution analysis of DASEs reveals cell-type-specific splicing programs with functional relevance (A) Heatmap of the top 100 cell-type-specific DASEs (adjusted *p* < 0.05, Δintron PSI ≥0.15) from the Camp et al., 2017 dataset, with enriched GO terms displayed on the right. (B) PSI values of selected DASEs projected onto a force-directed embedding of the iPSC differentiation trajectory (Camp et al., 2017). Left: cell states. Top-right and bottom-right: PSI values and corresponding gene expression levels. (C) Heatmap showing the top 100 cell-type-specific DASEs (adjusted *p* < 0.05, Δintron PSI ≥0.15), with enriched GO terms shown on the right. Cell types with fewer than 100 cells were excluded. Analysis is based on the Zhang et al. 2019 dataset. (D) PSI values of selected DASEs projected onto a UMAP embedding of cell clusters (Zhang et al., 2019). Left: cell identity. Top-right and bottom-right: PSI values and corresponding gene expression levels. (E) Force-directed layout of CD4^+^ and CD8^+^ T cell subtypes from the Zhang et al., 2019 dataset, including all donors. (F) PSI values of the *PTPRC* intron 3–5 across CD4^+^ and CD8^+^ T cell subtypes in the Zhang et al., 2019 dataset, including all donors. (G) Mean PSI values of *PTPRC* intron 3–5 across T cell subtypes, ordered from naive to activated states.

The heatmap demonstrated that each cell type displayed characteristic AS profiles throughout iPSC differentiation into hepatocytes (Figure 4A). In the undifferentiated state, iPSC-specific AS events were related to microtubule nucleation, DNA methylation, cell adhesion, nuclear migration, and other pathways, indicating that AS is involved in stem cell stem maintenance. In the DE and HE intermediate stages of differentiation, AS was mainly related to mitochondrial and cell death pathways, indicating that AS in this period was involved in energy supply and clearance of undifferentiated cells. At the end of differentiation, AS events were significantly enriched in pathways related to cell junction assembly, supramolecular fiber organization, triglyceride biosynthesis, cell adhesion, cell migration, and other processes, reflecting the characteristic functions of mature hepatocytes, such as barrier formation, secretory function, and lipid metabolism (Figure 4A). Moreover, the AS of most functionally relevant genes in mature hepatocytes was significantly altered at the end of differentiation (Figure S5A). We compared the changes in AS and gene expression of several representative genes during hepatocyte differentiation and found a weak correlation between the two, suggesting that AS operates independently of gene expression (Figure 4B).

*ESRP1* encodes an RNA-binding protein (RBP) that is involved in maintaining the epithelial phenotype of hepatocytes^41^ through AS. It has been shown that ESRP1 can regulate its own AS, a phenomenon commonly observed in RBPs.^42^^,^^43^ The *ESRP1* alternative intron is located in the 3UTR region of the gene, and its PSI changes from ∼40% before differentiation to ∼70% at the end of differentiation, suggesting that this event may be involved in regulating the RNA stability (Figures 4B and S5A). *ITGB1BP1* encode the integrin subunit β1-binding protein, which regulates hepatocyte adhesion and migration by modulating the β1 integrin signaling pathway.^44^ The mean PSI of introns 5–6 of *ITGB1BP1* is approximately 40% in iPSCs and decreases to 0 (indicating an increase in full-length isoforms) as differentiation progresses (Figures 4B and S5A).

Notably, intron 24–25 of *FN1* and intron 15 of *SEC31A* are specific to mature hepatocytes (Figures 4B and S5A). FN1-encoded fibronectin is an extracellular matrix protein involved in various signaling transductions. Synthesized by hepatocytes, fibronectin enters the bloodstream in a soluble form and contributes to clotting. Fibronectin has three alternative regions and theoretically can produce up to 20 isoforms.^45^ The *FN1* E25 encodes a complete type III domain, which is known as extra domains B + (EDB+). Studies have confirmed that both EDB+ and EDB− isoforms of fibronectin exist in cells, but they activate distinct signaling pathways, resulting in different cellular outcomes.^45^^,^^46^ Our study found that during iPSC differentiation, the conversion of FN1 from EDB+ to EDB− occurs, which may contribute to functional specialization in hepatocytes (Figure 4B). *SEC31A*, a component of COPⅡ coated vesicle, plays a crucial role in the formation and budding of endoplasmic reticulum vesicles. Studies have shown that *SEC31A* AS forms different isoforms that regulate the secretion of large and small cargo in the cell through lipid transport.^47^ Our analysis revealed that that inclusion of intron 15 in *SEC31A* increases during differentiation into IH and MH, suggesting its involvement in enhanced lipid metabolism and active secretion in hepatocytes (Figure 4B).

In addition to hepatocyte differentiation, we observed cell type-specific AS patterns across five leukocyte subtypes (Figure 4C). These splicing events are closely associated with cell lineage. CD4 and CD8 cells originate from T cell precursors, while both T cell precursors and NK cells derive from the common lymphoid progenitor. Macrophages differentiate from monocytes, which both originate from myeloid precursor cells. Our analysis revealed that AS patterns are more similar among closely related cell types (Figure 4C). GO analysis revealed significant enrichment of pathways related to actin or stress fiber assembly in four cell types: CD4, CD8, NK cells, and macrophages. These findings suggest a potential role for AS in immune cell activation and migration (Figure 4C). Other GO pathways identified are also closely related to the core functions of immune cells. For example, positive regulation of GTPase activity pathway facilitates the transport and anchoring of lysosome-associated cytotoxic granules by reorganizing the actin cytoskeleton, enabling their polarization toward the target cell contact site.^48^ Moreover, monocytes can directly kill pathogens by releasing proteases, myeloperoxidase, and antimicrobial peptides through the neutrophil degranulation pathway.^49^

We showed highly cell type-specific AS events in leukocytes (Figure S5B), with AS in representative genes showing weak correlation with expression levels (Figure 4D). The *FYB* gene encodes a T cell receptor (TCR) signal^16^ transduction adaptor protein, and phosphorylation in its N-terminal regulatory domain is required for binding and activating RhoA GTPase.^50^^,^^51^ PSI of *FYB* intron 1 was 10% in myeloid cells, but reached 83% and 71% in NK and CD8 cells, respectively (Figure 4D). Although the role of intron 1 in the N-terminal coding of *FYB* remains unclear, this event may represent a new pathway in cellular immune activation. SH3KBP1, which encodes CIN85, is lowly expressed in all leukocytes but exhibits significant AS in lymphocytes (Figure 4D). CIN85 functions as a key adaptor in TCR downstream signaling, and loss of *SH3KBP1* has been shown to result in excessive T cell proliferation and activation.^52^ Although *SH3KBP1* is known to generate multiple isoforms through AS,^53^ the specific roles of these isoforms in lymphocytes remain to be elucidated.

Another example is the *FGR* gene, a tyrosine kinase of the Src family required for NK cell activation through interaction with the adaptor protein MIST.^54^ *FGR* is highly expressed in monocytes, but its high PSI of intron 1 is almost exclusive to NK cells (Figure 4D). The seven FGR isoforms, which differ in 5′UTRs, encode the same protein,^55^ and the mechanism and function of NK-specific splicing at intron 1 remain unclear.

*PTPRC* encodes CD45 transmembrane protein tyrosine phosphatase, which is a pan-leukocyte marker. We observed that *PTPRC* was expressed across all leukocyte types, with slightly lower expression in macrophages. In NK cells, the PSI of *PTPRC* intron 3–5 was low, while it was elevated in CD4 and CD8 T cells, suggesting that the exon 3–5 skipped isoform is predominant in T cells (Figure 4D). Previous studies have shown that the full-length *PTPRC* isoform is characteristic of naive T cells, and the 4–6 skipped isoform is preferentially expressed in activated and memory T cells.^56^ We further analyzed the PSI of *PTPRC* intron 3–5 in T cell subtypes (Figure S5C). Consistent with previous reports, the PSI values of *PTPRC* intron 3–5 increased from naive T cells to activated T cells, especially in CD4 T cells (Figures S5D and S5E). Given the high heterogeneity of tumors, we extended our analysis to all donors in the Zhang et al. 2019 dataset, observing the same trend of transition from the skipped isoform to the full-length isoform, with a clear progression from naive to activated T cells (Figures 4E–4G). Notably, a separate study that sequenced PBMCs from healthy individuals using the 10× Genomics 5′ v.2 RNA-seq method also reported similar *PTPRC* splicing dynamics in T cell subtypes.^19^

### Computational resource evaluation

**Figure 5:**
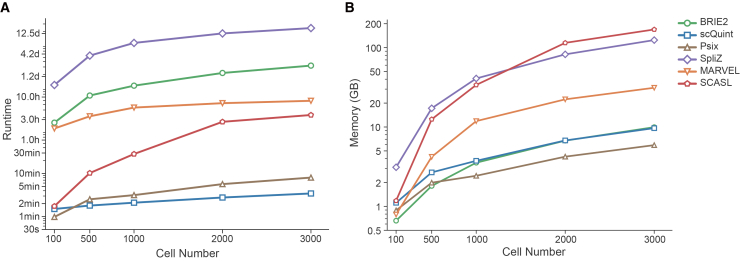
Computational resources consumed by the six methods (A and B) Computational time (A) and memory usage (B) of six methods for generating processed alternative splicing profiles across datasets with varying cell numbers.

Next, we evaluated the runtime and memory usage of the six AS analysis methods. To do so, we randomly sampled 100, 500, 1000, 2000, and 3000 cells and assessed computational performance on each subset. We found that scQuint and Psix completed processing 3000 cells within a few minutes (Figure 5A). In contrast, SCASL required over 3.8 h, BRIE2 took 53 h, and SpliZ required approximately 16 days to complete the same task. In terms of peak memory usage, BRIE2, scQuint, and Psix used approximately 1 GB of peak memory for 100 cells, increasing to around 9 GB for 3,000 cells (Figure 5B). In comparison, MARVEL required ∼30 GB at 3000 cells, while SpliZ and SCASL used 124 GB and 169 GB, respectively. Overall, scQuint demonstrated high scalability, with low computational cost in both time and memory, making it particularly suitable for large-scale single-cell AS analysis.

Beyond scalability, we further examined the effect of input feature selection on clustering performance. Unlike conventional scRNA-seq clustering, which typically selects highly variable genes, scQuint achieved optimal performance across datasets when using all detected splicing events as input (Figure S6D). A similar trend was observed for other methods (Figures S6A and S6C), where including all events consistently improved clustering outcomes. To assess the parameter robustness of clustering performance with respect to parameter selection, we further summarized BCI scores across all tested parameter combinations for each method. As shown in Figure S6B, scQuint and Psix exhibited relatively limited variation in BCI scores across different parameter settings, whereas SCASL showed substantially larger variability, with markedly reduced performance under certain configurations. Notably, even under less favorable parameter choices, scQuint maintained comparatively strong clustering performance, indicating a robust lower performance bound when parameters are not optimally tuned.

## Discussion

Due to the limitations of single-cell data, such as sparsity, research on AS at the single-cell level remains limited. Although several single-cell AS methods have been developed, a systematic evaluation of their performance has been lacking. In this study, we conducted the first comprehensive benchmarking of these methods using real single-cell datasets as ground truth, covering multiple tissues from both human and mouse. We found that scQuint consistently outperformed other methods in both cell subtype identification and DASE detection.

Using scQuint to analyze single-cell datasets from iPSC differentiation and tumor-infiltrating immune cells, we identified numerous cell-type-specific AS events. During hepatocyte differentiation, AS patterns exhibited dynamic transitions that closely aligned with functional specialization. Early stages were enriched in pathways related to microtubule nucleation and DNA methylation, while late stages showed enrichment in pathways related to cell junction organization and lipid metabolism. This is consistent with previous studies showing that liver remodeling after birth involves extensive post-transcriptional regulation that is both cell-type specific and temporally coordinated.^57^ In immune cells, we further identified the splicing dynamics of *PTPRC* during T cell activation, a process that bulk RNA-seq cannot resolve at the cell-type level.^58^^,^^59^ Together, our results fill an important gap in the evaluation of single-cell AS methods and demonstrate their utility in uncovering biologically meaningful splicing variation at cellular resolution.

For cell clustering using single cell AS profiles, annotation-free methods, such as scQuint and SCASL, have demonstrated superior performance in our analysis. These methods can detect all types of AS events, unlike BRIE2, which is limited to detecting only SE events,^25^ and they are also capable of discovering novel events. Specifically, scQuint utilizes the output from STARsolo, which performs a unified alignment across all samples using two-pass mode. In the first pass, splice sites detected across all samples are recorded and then used as reference information in the second pass.^60^ This unified alignment approach is likely to improve the accuracy of detecting both known and novel splice sites, providing a more comprehensive and consistent splice site annotation compared to methods like SpliZ and SCASL, which rely on separate alignments for each sample. In addition, scQuint’s intron-centric and annotation-free design focuses on relative intron usage within genes, which may mitigate the impact of dropout-driven sparsity in single-cell RNA-seq data and contribute to robust performance across datasets with varying sequencing depth. This is consistent with previous studies showing that incorporating intronic reads alleviates sparsity in both gene-level and splicing analyses.^61^ For DASE detection, scQuint consistently outperformed the other methods. In contrast, SCASL’s performance was compromised by redundancy in detecting both 5′ and 3′ splice events, as well as its inability to group events sharing the same 5′ or 3′ splice sites for comparison, leading to reduced accuracy.

Although scQuint demonstrates superior performance in both cell subtype identification and DASE detection, it also has certain limitations. First, this study benchmarks methods using real scRNA-seq datasets and biologically motivated tasks, emphasizing practical utility and biological relevance rather than performance under controlled simulation settings. In addition, scQuint cannot distinguish between different types of splicing events, such as SE, A3SS, and alternative 5′ splice sites. Moreover, splicing events that share the same 3′ end is grouped together, which may obscure the contributions of individual events. For accurate interpretation, Sashimi plots are often necessary to visualize splicing patterns in detail. Clustering performance in this study is evaluated using the BCI score, which quantifies concordance between splicing-based clusters and existing cell-type annotations derived from gene expression-based analyses. As such, this concordance-based evaluation is inherently conservative and may give lower scores when splicing-based clustering patterns differ from gene expression-derived cell-type labels. For the Tasic et al. dataset, we randomly selected a subset of cell types. Although different selections may affect cell-type separability and thus influence clustering results, we observe consistent trends across five independent datasets (Figure S2B), with top-performing methods maintaining their advantage. Notably, most existing single-cell AS analysis methods are designed for full-length scRNA-seq data, particularly from Smart-seq protocols. Accordingly, our benchmarking was also conducted on Smart-seq datasets, and the conclusions of this study should be interpreted within this experimental scope. However, the majority of currently available scRNA-seq data are generated using 10× Genomics 3′-end protocols. Due to their strong 3′ read bias and relatively low sequencing depth, 10× datasets are generally considered suboptimal for splicing analysis.^27^^,^^62^ In the future, developing new approaches that can robustly infer splicing events from such data, for example by leveraging deep learning or cross-platform transfer learning, will be an important avenue to enable large-scale single-cell splicing studies across diverse platforms.

### Limitations of the study

Our benchmarking focuses on full-length scRNA-seq datasets generated using Smart-seq-based protocols, which are currently the primary data type supported by most single-cell AS analysis methods. Consequently, the performance of these approaches on droplet-based platforms such as 10× Genomics is not evaluated in this study. In addition, clustering performance was assessed using the BCI score, a concordance-based metric that emphasizes agreement with existing cell-type annotations derived from gene expression. While this provides a standardized and practical framework for comparison, it may underestimate scenarios in which AS captures biologically meaningful cell populations that are not well represented by expression-based annotations.

## Resource availability

### Lead contact

Requests for further information and resources should be directed to and will be fulfilled by the lead contact, Quanlong Jiang (jiangquanlong@163.com).

### Materials availability

This study did not generate new unique reagents.

### Data and code availability

• Data: All datasets analyzed in this study are publicly available from the original publications and repositories. Accession numbers are listed in the key resources table.
• Code: All original code used in this study is publicly available at GitHub: https://github.com/QuanlongJiang/scAS_Benchmarking. The repository is listed in the key resources table.
• Other items: Any additional information required to reanalyze the data reported in this paper is available from the lead contact upon request.

## Acknowledgments

This study was supported by grants from the Jiangsu Provincial Medical Key Discipline (Laboratory) Cultivation Unit (JSDW202249) and the Young and Middle-aged Academic Leaders of Jiangsu Qing-Lan Project (2024) to G.L., the Nantong Science and Technology Program (no. JC2024024) to J.S., and the 10.13039/100016806Natural Science Foundation of Nantong City (JC2023114) to D.Z. We also thank Yaqiang Cao for helpful suggestions.

## Author contributions

J.S. and Q.J. conceived the project. J.S. and Q.J. designed the framework and performed data analysis. J.S. and Q.J. wrote the manuscript with input from all authors. G.L. and L.X. helped collect the data. D.Z. contributed to manuscript revision. All authors read and approved the final manuscript.

## Declaration of interests

The authors declare no competing interests.

## STAR★Methods

### Key resources table

**Table:** 

REAGENT or RESOURCE	SOURCE	IDENTIFIER
**Deposited data**		

Hepatocyte-like lineage	Camp et al., 2017	GEO: GSE81252
Primary Colorectal Cancer	Wang et al.^62^	SRA: SRP113436
Kidney	Tabula Muris, 2020	GEO: GSE132042
HCC lymphocyte	Zhang et al.^31^	HRA: HRA000069
Neocortical areas	Tasic et al.^33^	GEO: GSE115746
Primary human myoblasts	Trapnell et al., 2014	GEO: GSE52529
Motor neuron lineage	Song et al.^20^	GEO: GSE85908
Embryonic stem cells	Aleksandra et al., 2015	ArrayExpress: E-MTAB-2600

**Software and algorithms**		

scQuint	Benegas et al.^26^	https://github.com/songlab-cal/scquint
BRIE2	Huang and Sanguinetti^25^	https://github.com/huangyh09/brie
Psix	Buen Abad Najar et al.^29^	https://github.com/lareaulab/psix
SpliZ	Olivieri et al.^30^	https://github.com/salzman-lab/SpliZ
MARVEL	Wen et al.^28^	https://wenweixiong.github.io/MARVEL_Plate.html
SCASL	Xiang et al.^27^	https://github.com/xryanglab/SCASL
STAR	Dobin et al., 2013	https://github.com/alexdobin/STAR
rMATS-turbo	Shen et al., 2014	https://github.com/Xinglab/rmats-turbo
LeafCutter	Li et al., 2018	https://github.com/davidaknowles/leafcutter
scAS_Benchmarking	This paper	https://github.com/QuanlongJiang/scAS_Benchmarking

### Experimental model and study participant details

This study analyzed publicly available datasets from previously published studies involving human and mouse samples. Detailed information regarding sample sources, sequencing platforms, and accession numbers is provided in the Key Resources Table and original publications. No new human participants or animal experiments were involved in this study.

Sex information was not consistently available across all publicly available datasets analyzed in this study; therefore, sex-specific effects were not evaluated.

No cell lines were generated or experimentally maintained in this study.

### Method details

#### Ground-truth datasets

Five scRNA-seq datasets were used for clustering evaluation, including Camp et al. 2017 (iPSC to hepatocyte differentiation), Wang et al. 2021 (primary colorectal cancer), Tabula Muris 2018 (kidney tissue), Zhang et al. 2019 (HCC-infiltrating lymphocytes from donor D20170327), and Tasic et al. 2018 (10 randomly selected neocortical cell types, subsampled to reduce computational burden and ensure comparability across datasets). Five datasets from three studies were used to evaluate the DASE analysis. In the Trapnell et al., 2014 dataset, skeletal myoblasts differentiated under low-serum conditions for 24, 48, and 72 h were compared to the undifferentiated 0-h state. The Song et al., 2017 dataset compared iPSCs with motor neurons. The Aleksandra et al., 2015 dataset compared mouse embryonic stem cells cultured in serum versus a2i conditions.

#### Raw data processing

Adapter trimming was performed on raw FASTQ files using Trimmomatic (v0.39). Reads were aligned to the GRCh38 or GRCm38 reference genome using STAR (v2.7.11b) with --twopassMode Basic and default parameters. For scQuint and Psix, reads from all single cells were jointly aligned using STARsolo. Other methods performed alignment separately for each single cell using STAR.

#### Parameter settings for single-cell AS analysis methods

In our study, we evaluated the performance of 6 methods designed for single-cell RNA splicing. The parameters used for each of these algorithms were determined as follows.

1. BRIE2: We followed the instructions provided on the BRIE2 Website via: https://brie.readthedocs.io/en/latest/index.html.
2. scQuint: We followed the instructions provided on the scQuint GitHub repository via: https://github.com/songlab-cal/scquint.
3. Psix: We followed the instructions provided on the Psix GitHub repository via: https://github.com/lareaulab/psix
4. SpliZ: Input files for SpliZ were generated following the guidelines provided in the SICILIAN GitHub repository via: https://github.com/salzman-lab/SICILIAN/and SpliZ was executed according to the instructions in the GitHub repository via: https://github.com/salzman-lab/SpliZ.
5. MARVEL: We followed the guidelines provided on the MARVEL GitHub repository: https://wenweixiong.github.io/MARVEL_Plate.html.
6. SCASL: We followed the instructions provided on the SCASL GitHub repository via: https://github.com/xryanglab/SCASL

For more detailed usage instructions and analysis scripts used in this study, please refer to our GitHub repository: https://github.com/QuanlongJiang/scAS_Benchmarking.

Other methods. We next discuss other papers on this topic and clarify our reasons for not including them in the analysis. We did not consider methods that detect only group-level DAS by pooling reads from multiple cells without aiming to output high-confidence single-cell PSI estimates.^63^ We also excluded methods that calculate junction read counts or transcript abundances without computing junction usage ratios,^64^^,^^65^^,^^66^^,^^67^ require spike-in controls for analysis,^68^ focus on characterizing splicing modalities,^20^ or are primarily intended for visualizing splicing patterns.^69^

#### Parameter settings for bulk AS methods

rMATS-turbo v4.3.0 and LeafCutter were used for bulk AS analysis according to their official documentation (https://github.com/Xinglab/rmats-turbo, https://davidaknowles.github.io/leafcutter/) with default parameters.

#### Single-cell dimensionality reduction using alternative splicing events

Dimensionality reduction and clustering outcomes can be influenced by parameter choices, which may introduce bias when comparing methods. To minimize such confounding effects, we evaluated combinations of key parameters, including highly variable splicing features (top 2000, top 5000 or all events), number of neighbors (10, 15, 20, 30, 40), PCA components (5, 7, 9, 10, 15, 20), and Leiden clustering resolution (0.6–1.1). For each parameter combination, we calculated the BCI score and identified, for each method, the parameter set that achieved the highest score. This procedure ensured that all methods were assessed under their respective optimal yet comparable conditions.

### Quantification and statistical analysis

#### Benchmark metrics

Accuracy was calculated as the proportion of correctly predicted cells among all cells. For each cluster, the predicted label was assigned as the most frequent true cell type, and all cells within the cluster were assigned this label. ARI and NMI were calculated to assess the concordance between the predicted labels and the known cell-type annotations. The silhouette score was used as an unsupervised metric to evaluate the cohesion and separation of clusters based on pairwise distances between cells. To provide an integrated measure of clustering performance, we calculated the BCI score, defined as the mean of four metrics including accuracy, ARI, NMI, and silhouette score.

#### Concordance analysis between single-cell and bulk DASE results

To assess the agreement between differential splicing detection results from matched single-cell and bulk RNA-seq datasets, we calculated the AUCC based on the top-ranked events. Events were ranked within each dataset based on the statistical significance of differential splicing. For increasing values of *k* (e.g., top 1, top 2, …, top *k* events), we computed the overlap between the top-*k* event lists from single-cell and bulk analyses. This yielded a concordance curve representing the cumulative number of overlapping events as *k* increases. AUCC was obtained by summing the sizes of these intersections and normalizing by the maximum theoretical overlap, calculated as *k* × (*k* + 1) ⁄ 2. Unless otherwise specified, *k* was set to 200 or 100.

#### Cumulative recall benchmarking for experimentally validated events

We manually curated 9 differentially AS events that were experimentally validated by fluorescence *in situ* hybridization or quantitative PCR. For each method, the top 500 DASEs ranked by statistical significance were evaluated for their ability to recover these validated events. Cumulative recall curves were generated, where the x axis (rank 1–500) represents the significance-ranked DASEs and each increment along the y axis corresponds to an additional validated event recovered. Methods that achieve full recovery using fewer top-ranked predictions are considered to have better early recall performance.

#### Scalability analysis

The scalability analysis was conducted on a supercomputing platform consisting of a CPU computer cluster with one AMD Ryzen Threadripper 3960X 24-Core Processor (2.2 GHz base frequency, 3.8 GHz max frequency, 16 MB L3 cache and 48 CPU threads), 251 GB of DDR4 memory. In the evaluation of computer resources, we measured the time and memory usage from the mapped files by STAR to the generation of splicing profile matrices used for downstream analysis. The DAS analysis step was not included, as it typically completes within a few minutes and consumes minimal memory.

## Supplemental information

Document S1. Figures S1–S6

Table S1. Detailed information on scRNA-seq datasets used in this study This document contains eight sheets, each corresponding to one scRNA-seq study, listing the sample IDs together with the associated cell IDs and cell type annotations used in this study.

Table S2. Parameter sweep for clustering evaluation with optimal settings determined by BCl score Each row lists the method, dataset, tested parameters (number of features, number of neighbors, PCA components, Leiden clustering resolution), and corresponding evaluation metrics (accuracy, ARI, NMI, silhouette score, and BCI).
