# Supplementary material for: Comprehensive assessment of alternative splicing analysis methods for single-cell RNA-seq
Source: iScience. 2026 May 22;29(6):116090. doi: 10.1016/j.isci.2026.116090 (PMC13217931; doi:10.1016/j.isci.2026.116090)
Supplement: Document S1. Figures S1–S6 [file mmc1.pdf]

iScience, Volume *29*

Supplemental information

Comprehensive assessment of alternative splicing analysis methods for single-cell RNA-seq

Quanlong Jiang, Guicai Li, Lingyan Xing, Dongmei Zhang, and Junjie Sun

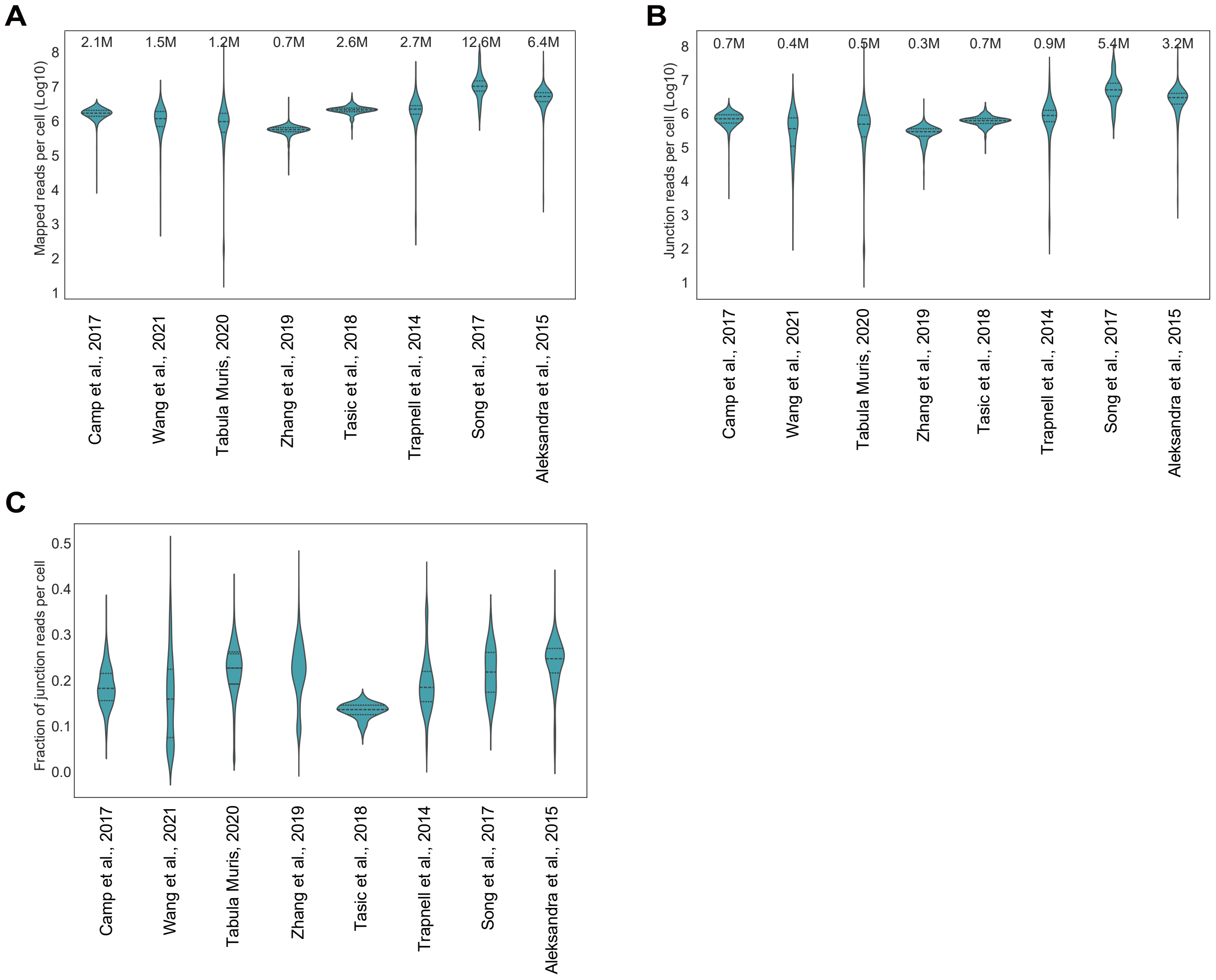

Fig. S1. Summary of sequencing read metrics across datasets. (A–C) Violin plots showing, for each dataset, the distribution per cell of (A) mapped reads, (B) junction reads, and (C) fraction of junction reads. Median values are indicated above each plot.

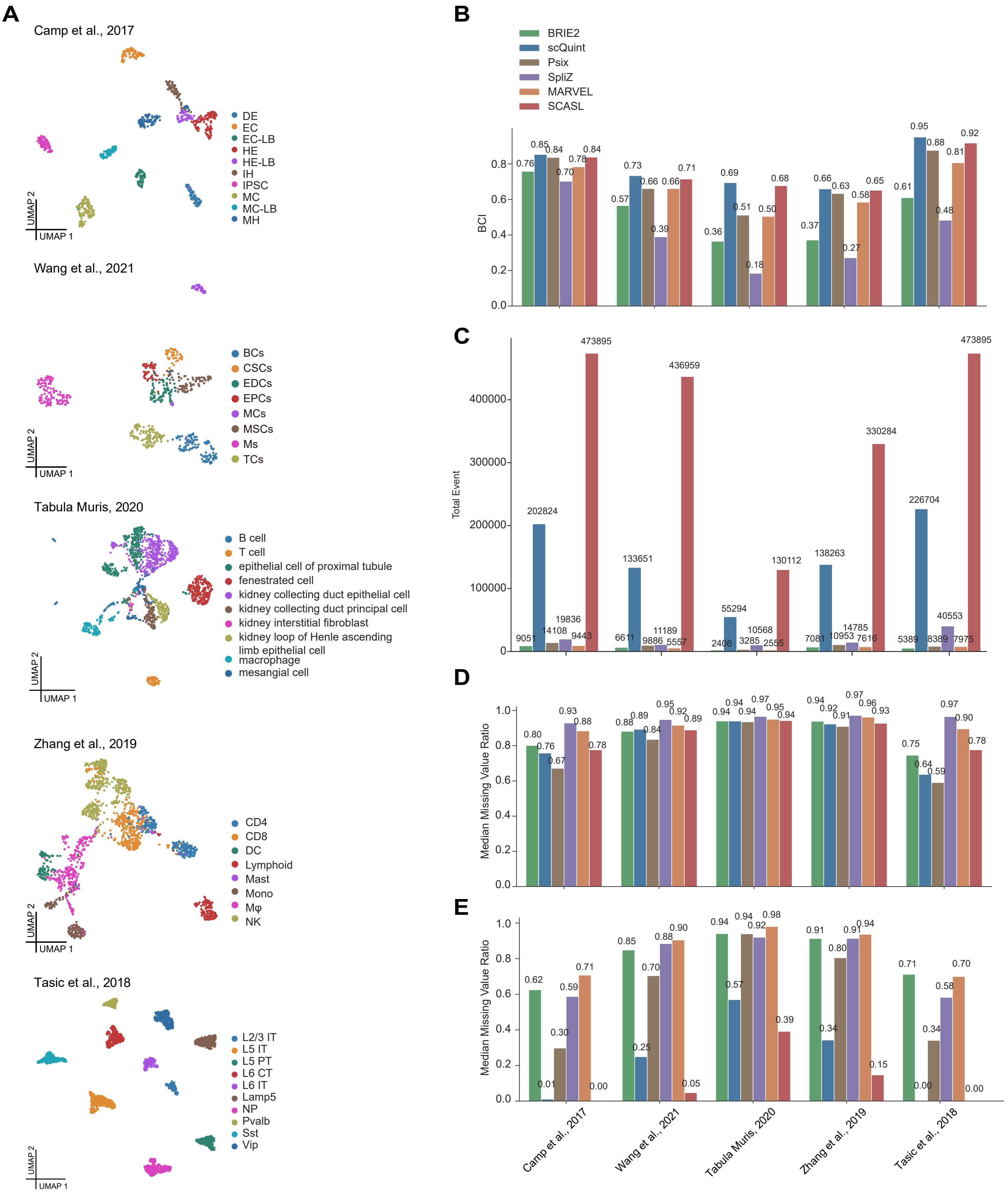

Fig. S2. Comparison of detected events, performance, and sparsity across AS methods. (A) UMAP visualization of five datasets based on gene expression, with clusters annotated by cell types. Datasets include Camp et al., 2017 (iPSC differentiation), Wang et al., 2021 (tumor-associated cells), Zhang et al., 2019 (immune cells), and Tasic et al., 2018 (neuronal subtypes), with detailed cell type annotations as indicated. (B) BCI scores of each method separated by dataset. (C) Total events detected by each method applied for dimensionality reduction on each dataset. (D) Median missing value ratio of events detected by each method, shown separately by dataset. (E) Median missing value ratio of the top 5,000 events with the least missing values, detected by each method, shown separately by dataset.

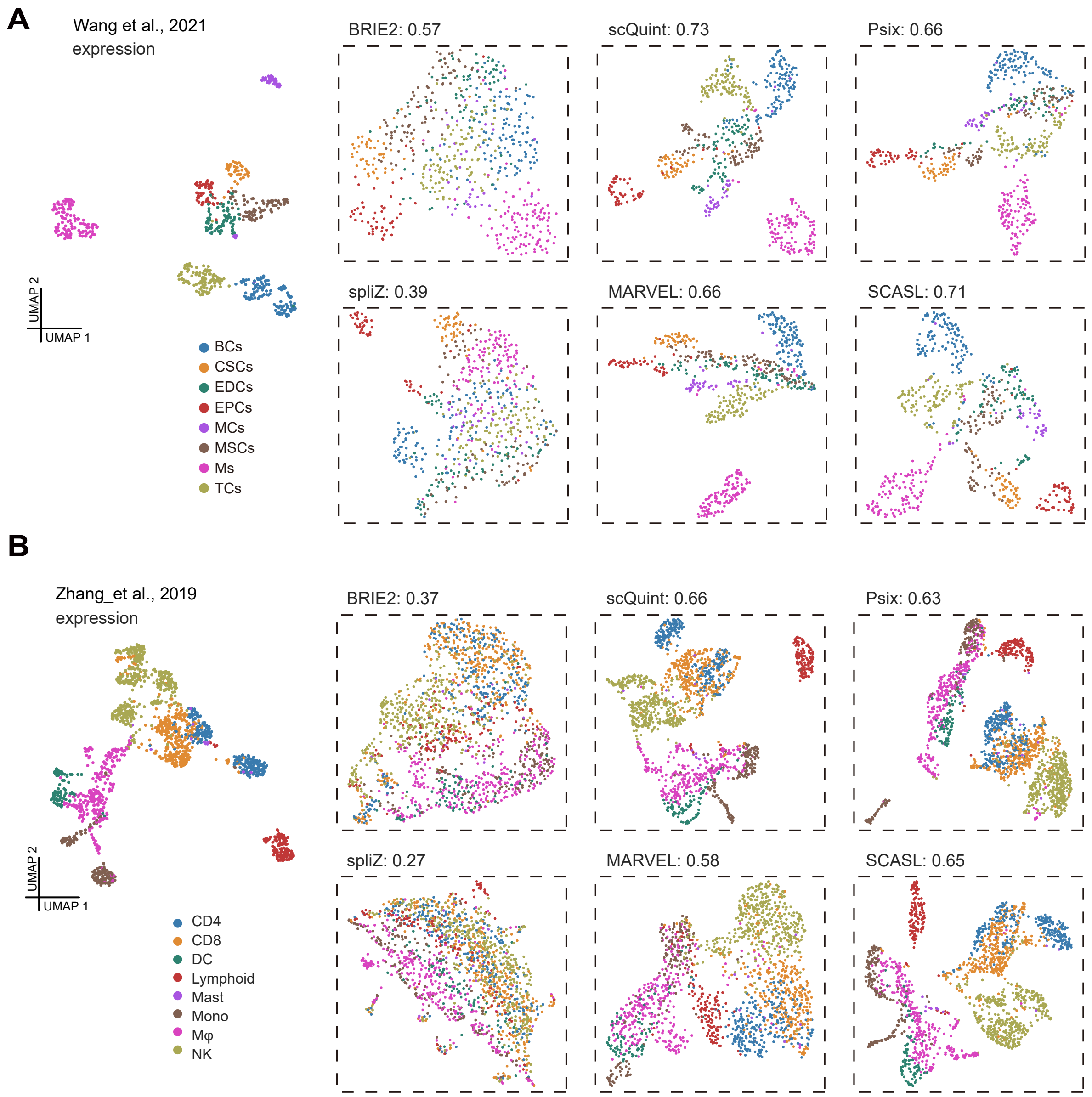

Fig. S3. Clustering evaluation of AS methods via UMAP across datasets. (A–B) UMAP embeddings based on alternative splicing profiles from the Wang et al. 2021 (A) and Zhang et al. 2019 (B) datasets demonstrate the discriminative ability of each method. Expression-based clustering is provided as a reference. BCI scores are shown alongside each method.

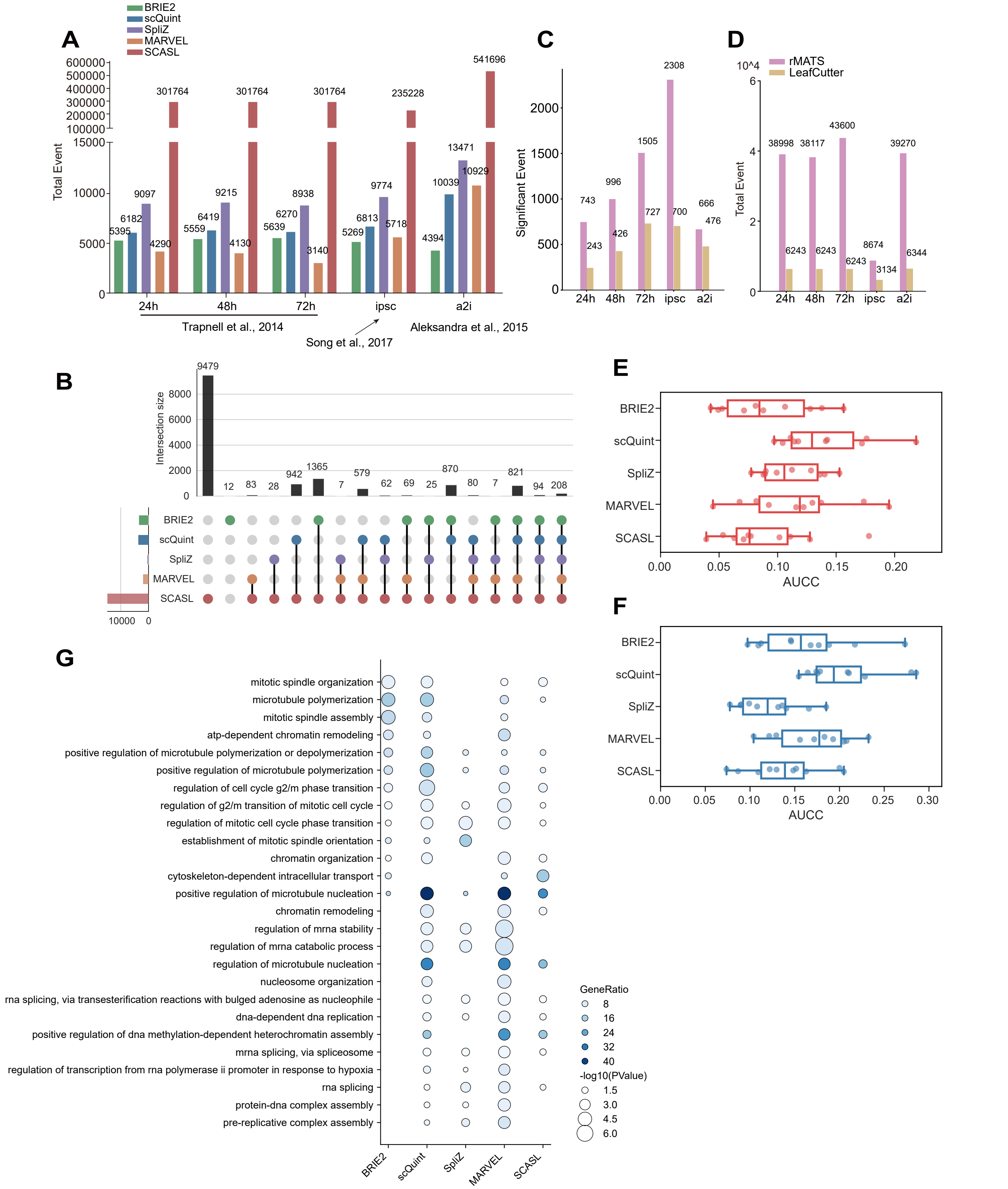

Fig. S4. Extended analysis of alternative splicing event detection across methods. (A) Bar plots showing the number of events used for DAS analysis y each single-cell method. For scQuint, events sharing the same 3′ splice site were grouped and analyzed together. (B) Upset plot showing overlaps among all detected alternative splicing events across different methods, based on data from Song et al., 2017. (C–D) Detection results from two bulk RNA-seq methods for alternative splicing analysis. (C) Number of significant differential splicing events. (D) Total number of detected splicing events. Notable differences between rMATS-turbo and LeafCutter may reflect their distinct definitions of alternative splicing events and statistical frameworks. (E–F) AUCC based on the top 100 ranked splicing events per method, evaluated at the event level (E) and gene level (F), across matched bulk–single-cell datasets. Boxplots represent median, interquartile range (IQR), and whiskers extending to 1.5×IQR.(G) GO enrichment analysis of the top 200 ranked splicing events per method, based on Song et al., 2017 dataset comparing iPSCs with MNs. Each bubble represents a GO term enrichment result, with the size indicating enrichment significance (−log10P-value) and color intensity representing the gene ratio.

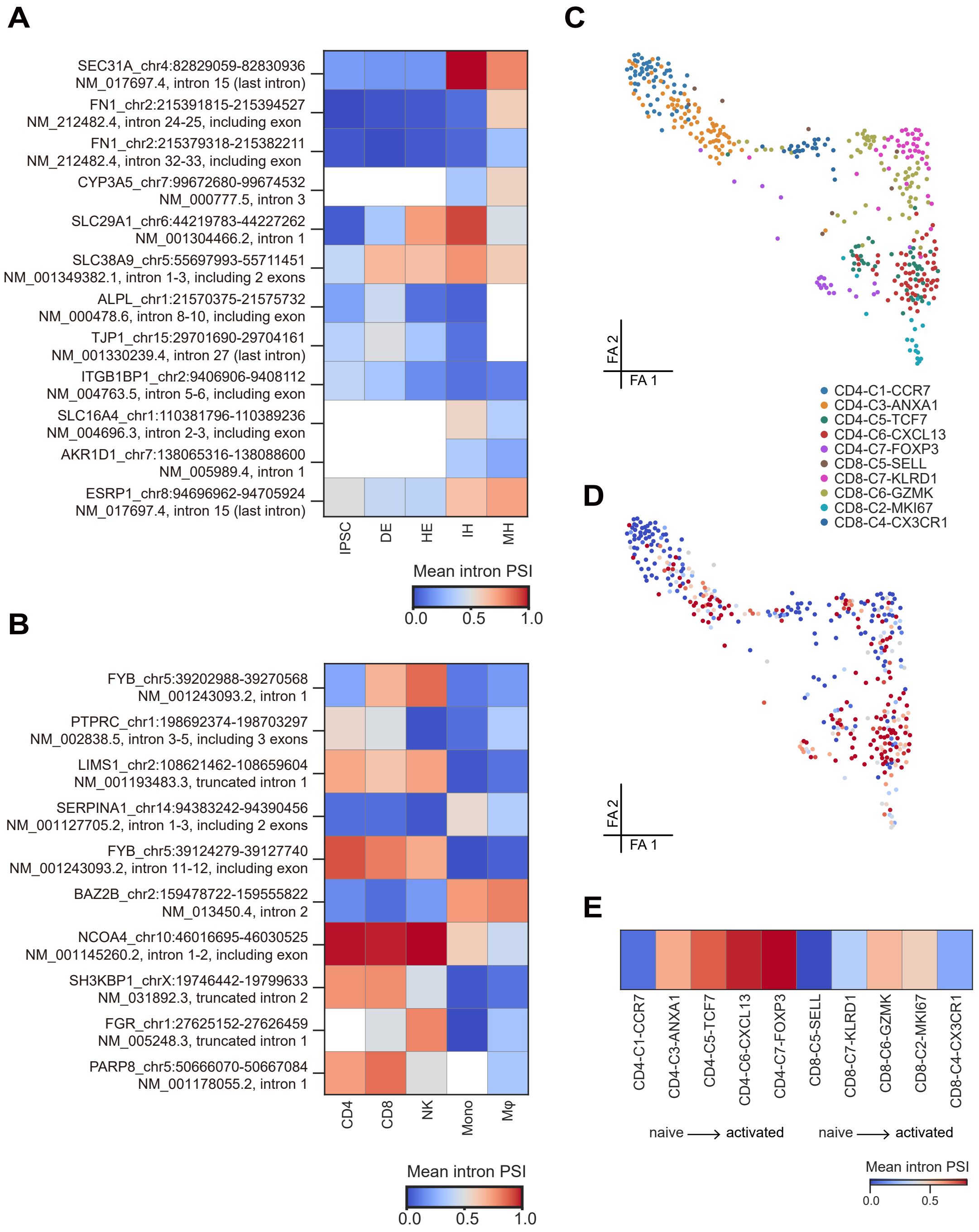

Fig. S5. Visualization of splicing dynamics across cell types. (A–B) Heatmaps of representative DASEs from the Camp et al., 2017 dataset (A) and the Zhang et al., 2019 dataset (B). Cell types with fewer than 10 cells are left blank. (C) Force-directed layout of CD4+ and CD8+ T cell subtypes from a single donor in the Zhang et al., 2019 dataset. (D) PSI values of the PTPRC intron 3–5 across CD4+ and CD8+ T cell subtypes from the same donor. (E) Mean PSI values of the PTPRC intron 3–5 across T cell subtypes, ordered from naïve to activated states, based on the same donor.

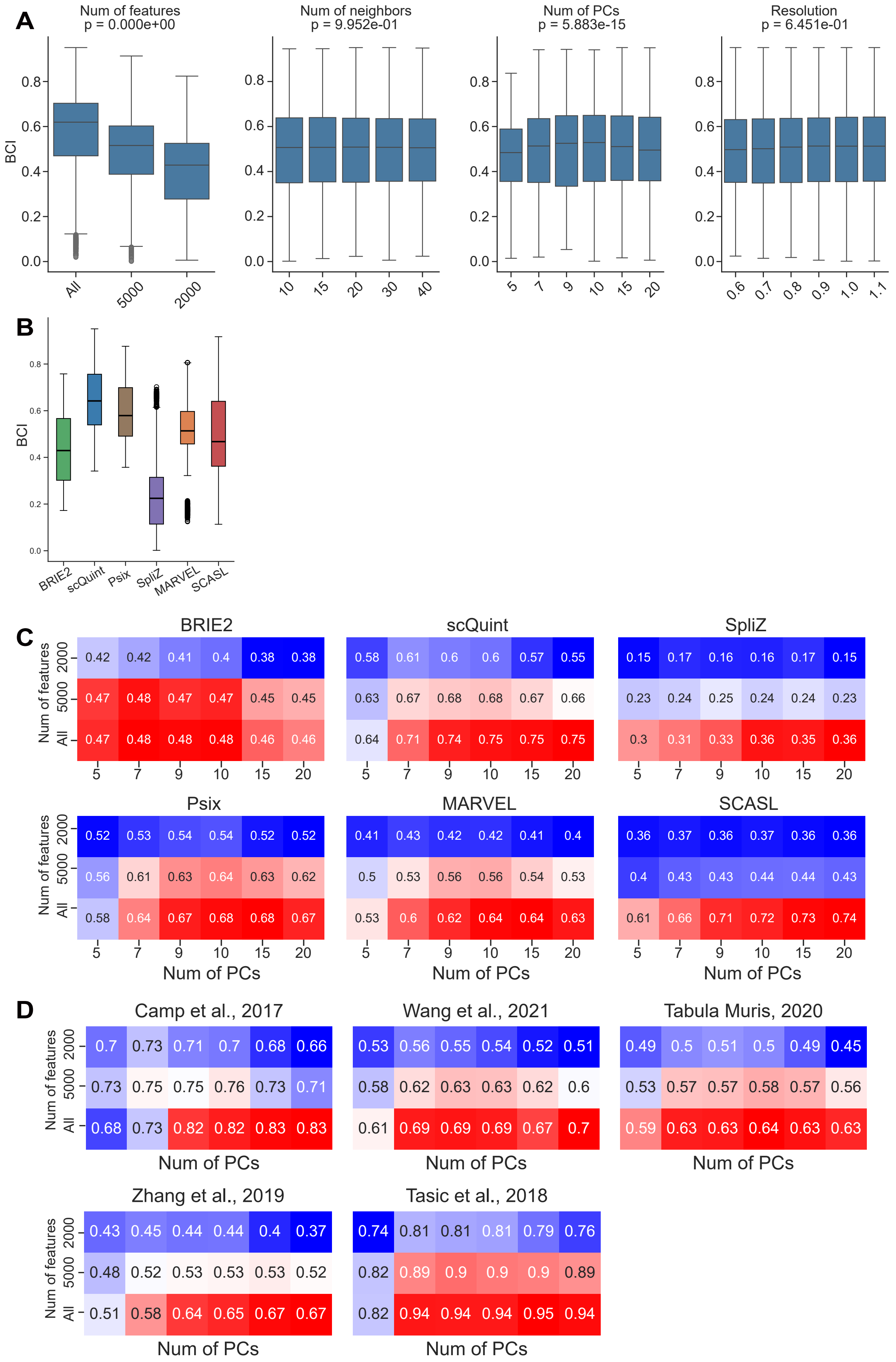

Fig. S6. Effects of parameter settings on clustering performance. (A) Boxplots showing BCI scores influenced by different parameter settings: the number of top variable splicing events (2,000, 5,000, or all events), the number of neighbors, the number of principal components, and the clustering resolution (from left to right). (B) Boxplots showing the overall range of BCI score variation for each software across different parameter combinations and datasets. Box plots show median (center line), interquartile range (box), and 1.5× IQR (whiskers). Points represent individual datasets.(C) Heatmap showing the influence of the combination of splicing event number and principal component number on BCI scores for each method. (D) Heatmap showing the influence of the combination of splicing event number and principal component number on scQuint BCI scores across datasets.
